# Cryptic or Silent? The Known Unknowns, Unknown Knowns, and Unknown Unknowns of Secondary Metabolism

**DOI:** 10.1128/mBio.02642-20

**Published:** 2020-10-20

**Authors:** Paul A. Hoskisson, Ryan F. Seipke

**Affiliations:** a Strathclyde Institute of Pharmacy and Biomedical Sciences, University of Strathclyde, Glasgow, United Kingdom; b Faculty of Biological Sciences, Astbury Centre for Structural Molecular Biology, University of Leeds, Leeds, United Kingdom; McMaster University

**Keywords:** *Actinobacteria*, natural products, secondary metabolism, specialized metabolism, *Streptomyces*

## Abstract

Microbial natural products, particularly those produced by filamentous *Actinobacteria*, underpin the majority of clinically used antibiotics. Unfortunately, only a few new antibiotic classes have been discovered since the 1970s, which has exacerbated fears of a postapocalyptic world in which antibiotics have lost their utility. Excitingly, the genome sequencing revolution painted an entirely new picture, one in which an average strain of filamentous *Actinobacteria* harbors 20 to 50 natural product biosynthetic pathways but expresses very few of these under laboratory conditions. Development of methodology to access this “hidden” biochemical diversity has the potential to usher in a second Golden Era of antibiotic discovery. The proliferation of genomic data has led to inconsistent use of “cryptic” and “silent” when referring to biosynthetic gene clusters identified by bioinformatic analysis. In this Perspective, we discuss this issue and propose to formalize the use of this terminology.

## Abstract (precis)

Microbial natural products, particularly those produced by filamentous *Actinobacteria*, underpin the majority of clinically used antibiotics. Unfortunately, only a few new antibiotic classes have been discovered since the 1970s, which has exacerbated fears of a postapocalyptic world in which antibiotics have lost their utility. Excitingly, the genome sequencing revolution painted an entirely new picture, one in which an average strain of filamentous *Actinobacteria* harbors 20 to 50 natural product biosynthetic pathways but expresses very few of these under laboratory conditions.

## PERSPECTIVE

Almost a century of research and industrial activity has earned *Streptomyces* species and other filamentous *Actinobacteria* a well-deserved reputation for their ability to produce a vast array of natural products, many of which have found utility in the clinic. Consequently, these microbes have been an important resource for the pharmaceutical industry. Indeed, some compounds that were developed into drugs (e.g., clavulanic acid, chloramphenicol, spectinomycin, ivermectin, amphotericin, and nystatin) are now considered essential medicines by the World Health Organization (1). The Golden Age of antibiotic discovery came to an end in the 1970s, because existing pipelines for new molecules ran dry and rediscovery of known compounds was common (the so-called “dereplication problem”). Generally, natural product discovery programs were replaced with combinatorial chemistry platforms that produced large chemical libraries for high-throughput screening, which largely failed to produce new lead compounds. In the longer term, industrial antibiotic discovery programs were no longer financially viable and Big Pharma divested from discovery programs (2). This reduction in antibiotic discovery capacity has been against a backdrop of increasing antimicrobial-resistant infections within the clinic and community to the point that there is now an urgent need to discover and develop novel antimicrobials for the clinic.

It is not all doom and gloom, however; we are living in the “Genomics Age” of antibiotic discovery, which began in 2002 with the sequencing of the first *Streptomyces* genome, Streptomyces coelicolor A3(2) (3). Analysis of this genome sequence identified a greater number of natural product biosynthetic gene clusters (BGCs) than the organism was known to produce. A further 18 BGCs were identified, but at the time, only four compounds had been observed in the laboratory. This exciting revelation gave rise to the notion that on the whole, secondary metabolism in S. coelicolor A3(2) is relatively inactive in a laboratory setting, with most molecules detected in laboratory culture having been previously identified and linked to biosynthetic gene clusters (BGCs) (Known Knowns).

This was established as a widespread phenomenon, rather than a quirk of S. coelicolor, when the genomes of Streptomyces avermitilis, Streptomyces griseus, and Streptomyces scabies were sequenced and analyzed (4–6). Since these genomes were “mined” for secondary metabolites, it has become apparent that the genomes of *Actinobacteria* represent a vast repository of novel natural product chemistry. For example, a study of 830 actinobacterial genomes identified >11,000 natural product BGCs that represent >4,000 chemical families (with chemical families being compounds with similar chemical backbones that may possess similar physical and chemical properties [7]) which tells us that there is vast chemical space out there in nature. While there are rare cases of similar or identical molecules being produced by evolutionarily distinct BGCs (8, 9), the prevalence of this scenario will remain unknown until high-throughput chemical analysis catches up with genomics. The newfound abundance of genomic data has resulted in an explosion of methods to computationally mine actinobacterial genomes for natural product BGCs such as antiSMASH, PRISM, EvoMining, and DeepBGC (10–13), which can be linked to natural product databases (MIBiG, antiSMASH database, and Natural Product Atlas) (14–16). The prioritization of which BGCs to study remains a challenge in the field, but the wealth of data available indicates that there are many new compounds out there to be discovered (17).

The increase in genome sequencing and genome mining studies has, however, led to inconsistency in the terminology used to refer to the “hidden treasures” encoded by the genomes of natural product-producing organisms. In the literature, the use of “cryptic” and “silent” has become pervasive, and these terms are often used interchangeably to describe natural product BGCs for which the genes encoding the enzymes for biosynthesis have been identified but for which no product has been observed during laboratory culture. Inconsistent use of these terms is confusing, especially for newcomers to the field. In this perspective, we disambiguate “silent” and “cryptic” and propose rules for their use when applied to natural product BGCs.

“Cryptic” is derived from the Greek *kruptós* meaning “hidden” and is used widely in biology to describe well-camouflaged species (https://en.wiktionary.org/wiki/cryptic) or where two or more species have been misclassified as a single taxon due to being morphologically indistinguishable but are genetically distinct (cryptic species) (18). In microbiology the use of “cryptic” to describe plasmids that do not encode any known beneficial traits (19) is found regularly, but this perhaps reflects our poor understanding of the role these plasmids play in the ecology of many organisms, rather than them not being beneficial. “Silent” is a straightforward term that most are familiar with. For instance, “gene silencing” is well understood in RNA interference (RNAi) scenarios and “silent” is also used to describe gene conversion events in antigenic variation—a gene is present, but is not expressed, but can be expressed following a mutational event. These systems often reflect complicated and/or poorly understood gene regulatory systems. Thus, we propose that the term “cryptic” should be used for describing BGCs and/or natural products that are hidden or unknown and the term “silent” should be used only for describing BGCs that are not expressed.

**FIG 1:**
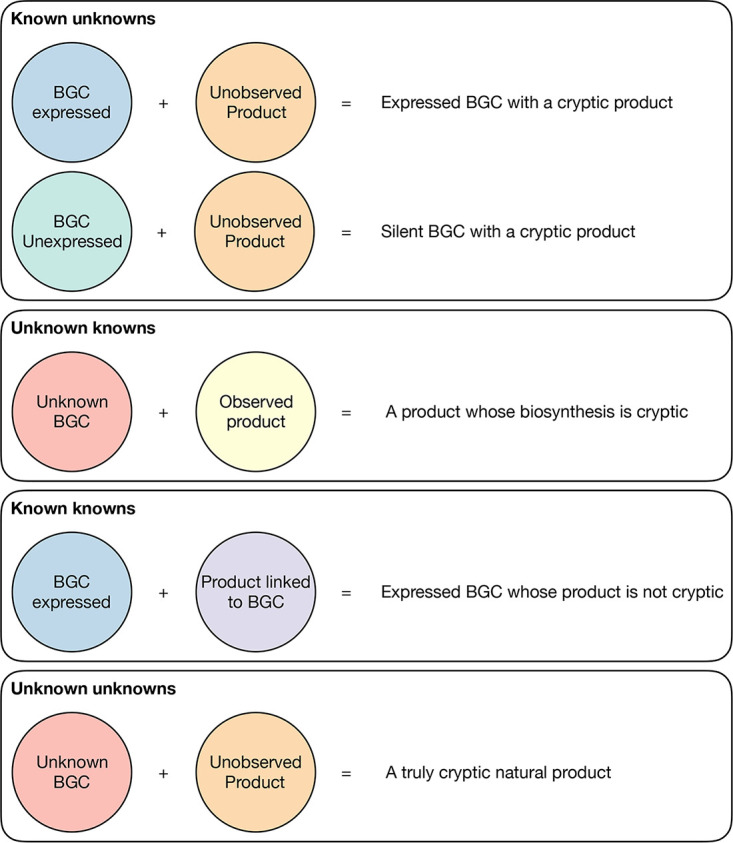
A summary of the description of various permutations of the cryptic or silent nature of secondary metabolism.

For instance, consider the following scenario and what the description of the BGC and product would be. One has bioinformatically analyzed a *Streptomyces* genome sequence, and a BGC has been identified that encodes a putative polyketide biosynthetic (PKS) system. Expression of the BGC has not been analyzed, so the use of “silent” is inappropriate. Future experimentation may establish that the PKS BGC is in fact unexpressed, which means the BGC should at this point be described as “silent.” By definition this means that the associated polyketide product is, of course, “cryptic,” because it cannot be produced if its biosynthetic genes are not expressed. We outline various other scenarios for the usage of cryptic and silent in Fig. 1.

The majority of the confusion has crept into literature when discussing the Known Unknowns and the Unknown Knowns. This is where it is important for the term “cryptic” to be used accurately. The use of “cryptic BGC” is appropriate when a natural product has been observed, but where its cognate BGC has not been experimentally identified, i.e., the compound is known, but its biosynthesis is cryptic (Unknown Knowns). Equally, the term “cryptic” can be appropriately used when expression of a BGC has been experimentally validated, but a product predicted from that BGC cannot be observed under laboratory culture conditions (Known Unknowns), i.e., the product of the BGC is cryptic.

Interestingly, it is the last category, the Unknown Unknowns, where the real hidden treasures likely lie. Currently, around one-third of all protein-encoding genes in bacterial genomes lack functional annotation (20). It is within these hypothetical protein-encoding genes where truly “cryptic BGCs” (Unknown Unknowns) may be located. These are completely novel BGCs, where the lack of functional annotation precludes them from being identified by the multitude of bioinformatic tools and natural product databases. The resulting secondary metabolites are also more likely to be missed when analyzing culture supernatants for bioactive molecules—we often look for the kinds of molecules that we are already familiar with. Again, screening natural product libraries for bioactivity does not investigate all “bioactivity space,” since it is usually confined to activity against a panel of well-known surrogate indicator organisms/cell lines (21).

Progress has been made in identifying these kinds of truly cryptic gene clusters using evolutionary mining methods to identify the expansion and repurposing of enzyme families from central metabolism. This is exemplified by the discovery of a BGC for an arsenopolyketide (22) and an unusual thiotemplated nonribosomal peptide synthetase (NRPS)-independent BGC encoding closthioamide biosynthesis (23, 24). The same evolutionary rationale applies to the duplication of some housekeeping genes, which confer resistance to the antibiotic, so-called target duplication (25). Broadly speaking, these evolutionarily guided approaches can expand the predictions of commonly used BGC prediction algorithms by up to 26% (12) and can potentially identify BGCs for natural products that have novel modes of action (25). With the exciting expansion of genomic, metagenomic, and metabolomic resources and their integration with both small- and large-scale functional studies (26), we owe it to ourselves to be precise in our terminology.
